# Multicenter, Multinational, and Multivendor Validation of an Artificial Intelligence Application for Acute Cervical Spine Fracture Detection on CT

**DOI:** 10.3390/diagnostics16020194

**Published:** 2026-01-07

**Authors:** Jinkyeong Sung, Peter D. Chang, Angela Ayobi, Martina Cotena, Mar Roca-Sogorb, Jinhee Jang, Daniel S. Chow, Yasmina Chaibi

**Affiliations:** 1 Applied Artificial Intelligence Research, University of California Irvine, 836 Health Sciences Road, Suite 4021, Irvine, CA 92617, USA; 2 Department of Radiology, Chung-Ang University Hospital, Chung-Ang University College of Medicine, Seoul 06591, Republic of Korea; 3 Department of Radiological Sciences, University of California Irvine, 1 Medical Plaza Dr, Irvine, CA 92697, USA; 4 Avicenna.AI, 375 Avenue du Mistral, 13600 La Ciotat, France; 5 Department of Radiology, Seoul St. Mary’s Hospital, College of Medicine, The Catholic University of Korea, Seoul 06973, Republic of Korea

**Keywords:** artificial intelligence, deep learning, cervical spine fracture, computed tomography

## Abstract

**Background/Objectives:** While previous studies have evaluated AI algorithms for cervical spine fracture (CSFx) detection on CT, many have lacked validation on diverse, multinational datasets or have focused primarily on overall case-level classification This study aimed to evaluate the performance of an AI application for acute CSFx detection in case-level classification, fracture localization, and spinal level labeling on multicenter, multinational, and multivendor CT data. **Methods:** Non-enhanced CTs were retrospectively collected from a U.S. teleradiology company, a French teleradiology company, and a U.S. university hospital. Four radiologists independently labeled the presence and location (including the spinal level) of acute CSFx to establish the reference standard. Per-case diagnostic performance, per-bounding box positive predictive value (PPV) for localization, and overall agreement of cervical vertebral level labeling of the AI were assessed. **Results:** A total of 155 patients (60.6 years ± 21.2 years, 104 men) with acute CSFx and 173 patients (51.9 years ± 22.7 years, 91 men) without acute CSFx were evaluated. Data were acquired using scanners from five manufacturers. For acute CSFx diagnosis, the AI achieved a per-case sensitivity of 90.3%, a specificity of 91.9%, an accuracy of 91.2%, an area under the receiver operating characteristic curve (AUC) of 0.91, and Matthews correlation coefficient of 0.82. Among 192 bounding boxes representing acute CSFx generated for 154 positive cases by the AI, 162 were true positives (per-bounding box PPV, 84.4%). Of the 186 bounding boxes for which the AI displayed cervical spinal level, 181 were labeled correctly (overall agreement, 97.3%). **Conclusions:** The AI application for detecting acute CSFx demonstrated high diagnostic performance on multicenter, multinational, and multivendor data, with high performance in fracture localization and spinal level labeling.

## 1. Introduction

Traumatic cervical spine fractures (CSFx) can lead to severe morbidity and mortality. In patients with concomitant spinal cord injury, the adverse outcome and mortality rate substantially increase [1,2]. CT, which has significantly higher sensitivity compared to plain radiographs in detecting CSFx, is commonly used as the primary imaging modality for cervical trauma patients in the emergency department [3]. However, CSFx may be overlooked due to the increasingly heavy workload on radiologists driven by rising demand for imaging as well as a growing elderly population with degenerative changes and osteoporosis [1,4].

Several studies have reported the development and validation of artificial intelligence (AI) algorithms for assisting in CSFx detection, which have shown variable performance [5,6,7,8,9,10,11]. Most studies were evaluated in limited settings, such as single-center, single-vendor datasets [5,6,7,9,11]. Furthermore, some studies established a reference standard based on consensus between radiologists and AI [5,7] or used consensus readings by radiologists, but only for a subset of the data [6,9]. These limitations reduce the robustness and generalizability of their conclusions. Although winning algorithms from the 2022 Radiological Society of North America (RSNA) AI challenge were evaluated on a multi-institutional and multinational expert-labeled dataset, performance assessment was limited to case-level classification [10].

The purpose of this study was to assess the capability of an AI-based application to detect acute CSFx through case-level classification, fracture localization, and spinal level labeling, leveraging multicenter, multinational, and multivendor CT data.

## 2. Materials and Methods

This retrospective, multicenter, multinational, and multivendor study was conducted in accordance with the Declaration of Helsinki. Before investigator assessments, all data were anonymized in accordance with HIPAA and GDPR requirements. Informed consent was waived in line with national legislation and institutional policies prior to data transfer. We followed the STARD (Standards for Reporting of Diagnostic Accuracy Studies) and CLAIM (Checklist for Artificial Intelligence in Medical Imaging) guidelines [12,13,14].

### 2.1. Data Collection

Prior to the study, a sample size estimation was performed to ensure adequate statistical power. Based on prior literature on AI algorithms for cervical spine fracture detection on CT [5,6,7,8,15], a lower bound of 80% for the 95% confidence intervals of both sensitivity and specificity was set as the target performance. The minimum number of cases required to meet this criterion was calculated using the PASS sample size software (version 20; Kaysville, UT, USA). Based on a binomial dichotomous endpoint for a single-sample study, a minimum of 137 cases with acute CSFx and 137 cases without acute fractures were necessary for this study.

Anonymized non-enhanced CT scans were retrospectively obtained from a U.S. teleradiology company (vRAD; Minneapolis, MN, USA), for which more than 80% of the data originate from trauma/emergency centers; a French teleradiology company (TeleDiag; Lyon, France), for which less than 50% of the data originate from trauma/emergency centers; and a U.S. university hospital (University of California, Irvine, level 1 trauma center).

**Figure 1:**
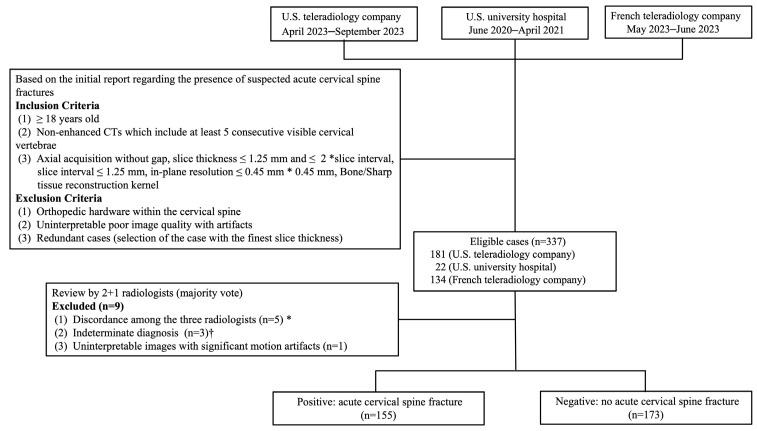
Flow diagram of the data selection process. * Three radiologists could not reach a consensus (for example, reader 1: positive, reader 2: negative, reader 3: indeterminate). † At least two radiologists defined the case as indeterminate. U.S. = United States.

Inclusion and exclusion criteria are listed in Figure 1. Non-enhanced CTs obtained from adult patients (≥18 years) that included at least five consecutive visible cervical vertebrae were included. Acquisition parameters for inclusion are as follows: axial acquisition without gap, slice thickness ≤ 1.25 mm and ≤2 xslice interval, slice interval ≤ 1.25 mm, in-plane resolution ≤ 0.45 mm × 0.45 mm, bone/sharp tissue reconstruction kernel. Exclusion criteria included orthopedic hardware within the cervical spine, uninterpretable poor image quality with artifacts, and redundant cases (selection of the case with the finest slice thickness). Sufficient cases were consecutively collected from each source at different times and classified as positive and negative according to initial radiologist reports. To achieve the required sample size, both positive and negative cases were retrieved from vRAD between April and September 2023 and from TeleDiag between May and June 2023. Since the number of positive cases obtained was insufficient, additional positive cases were retrieved from UCI between June 2020 and April 2021.

After radiologist review of 337 eligible cases, nine cases were excluded, as detailed in the results section. Finally, 155 patients with acute CSFx and 173 patients without acute fractures were included (Figure 1), which exceeded the minimum sample size requirement for statistical power. The dataset collected from the three centers in this study was not used for prior training.

### 2.2. AI Application

The AI application used was CINA-CSpine (version 1.0, Avicenna.AI, La Ciotat, France), which is designed to detect acute CSFx other than compression fractures. The device is FDA-cleared for prioritization and case-level classificatiaon and CE-marked for case-level classification, CSFx localization (with bounding boxes), and cervical spinal level labeling. The application has been commercially available in the U.S. since June 2024 and in Europe since April 2025.

**Figure 2:**
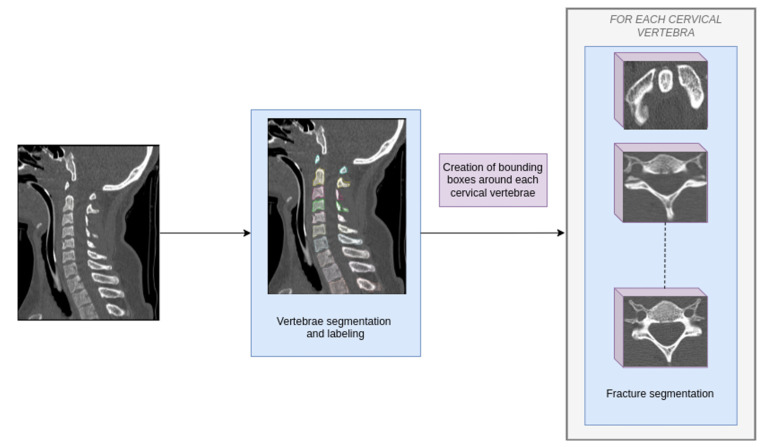
Schematic diagram of the high level overview of the two sequential algorithms used in the artificial intelligence application.

To develop CINA-CSpine, a cascade of fully 3D, task-focused U-Net CNNs was trained to detect CSFx [16]. This cascade comprises two sequential algorithms designed to mimic expert radiologists’ diagnostic reasoning: the first isolates each cervical vertebra within the CT images and the second identifies fractures within the isolated vertebrae. The combination of these steps enables precise, focused fracture detection (Figure 2).

The training dataset included 1338 CT studies, with 80% used for training and 20% for tuning. A total of 33% of the studies were positive cases with at least one CSFx. Model optimization employed a combination of Sørensen–Dice coefficient-based Cross-Entropy Loss and Generalized Dice Loss [17].

All the data were acquired in several U.S. and French centers and were adequately distributed in terms of patient and imaging characteristics. Validation on an internal test dataset of 221 multicenter CT studies achieved a sensitivity of 88.4% (95% CI: 81–94%) and specificity of 92.7% (95% CI: 86–97%).

### 2.3. Reference Standard

Two board-certified radiologists (a neuroradiologist with 20 years of experience and an emergency radiologist with 40 years of experience) independently labeled the presence (positive, negative, or indeterminate) and location, including spinal level, of acute CSFx (linear lucencies or displaced fractures) using multiplanar reconstruction mode. For each case, they selected the slices and planes (axial, sagittal, or coronal) where the fractures were most clearly visible and placed regions of interest (ROIs) encompassing the fractures. Cases were categorized as indeterminate if the radiologists could not determine the presence or absence of acute fractures. They also reported any confounding conditions (streak artifact, motion artifacts, presence of tumor, metastasis, bone diseases, Schmorl’s nodes, etc.), when observed. Cases deemed non-interpretable were rejected. Non-acute fractures and vertebral compression fractures were considered negative since they are not targeted by the AI application.

For case-level discrepancies (e.g., positive/negative, positive/indeterminate, negative/indeterminate), a third board-certified radiologist (an emergency radiologist with twenty years of experience) independently reviewed the case, and the final reference standard was determined by majority vote.

To evaluate the localization performance of bounding boxes generated by the AI application, in case of localization discrepancy despite per-case consensus (the case was classified as positive, but the ROIs were placed in different locations), a fourth board-certified radiologist (a musculoskeletal radiologist with 14 years of experience) independently reviewed the case. The final fracture location was determined by majority vote.

All radiologists were blinded to the clinical information, initial imaging reports, other radiologists’ labels, and AI outputs.

### 2.4. Statistical Analysis

Cohen’s kappa was calculated between the two initial radiologists who established the reference standard to evaluate the degree of inter-reader variability. Then, per-case sensitivity, specificity, and accuracy were calculated and compared between independent subgroups using the Chi-square test. The 95% confidence intervals (95% CI) were calculated using the exact binomial distribution (Clopper–Pearson) and the lower bounds of the two-sided 95% CI were compared to a performance goal of 80% for sensitivity and specificity [5,6,7,8,15]. Per-case positive predictive value (PPV), and negative predictive value (NPV) were also calculated. The area under the receiver operating characteristic (ROC) curve (AUC) was also computed at a per-case level. Furthermore, Matthews correlation coefficient (MCC) was calculated to assess binary classification of fracture vs. non-fracture [18,19,20].

Per-bounding box PPV was defined as the number of correctly detected and localized fractures divided by the total number of fractures detected by the AI application and displayed with bounding boxes. The accuracy of cervical spinal level labeling was assessed using overall agreement, calculated as the number of fractures labeled with the correct vertebral level divided by the total number of level-labeled fractures.

Per-case time-to-notification, defined as the time from the end of the DICOM reception to the end of processing, was also computed. For all statistical analyses, *p* < 0.05 was considered indicative of a statistically significant difference. All statistical analyses were performed using MedCalc Statistical Software (v22.023, MedCalc Software Ltd., Mariakerke, Belgium).

## 3. Results

### 3.1. Data Characteristics

Initially, a total of 337 cases were included in the study. Nine cases were excluded due to: discordance among three radiologists (*n* = 5), indeterminate diagnosis (*n* = 3), or significant motion artifacts (*n* = 1), resulting in a final cohort of 328 cases. Among the 328 cases included in the study, disagreements were observed between the first two radiologists for 25/328 (7.6%) cases, leading to a Cohen’s Kappa of 0.85 [95% CI: 0.79–0.90], which indicates a very good agreement. The data characteristics of 155 patients (mean age 60.6 ± SD 21.2 years) with acute CSFx and 173 patients (mean age 51.9 ± SD 22.7 years) without acute fractures are summarized in Table 1. For 14 cases, sex information was not available due to the anonymization process, which removed the associated DICOM tag. The data distribution by the CT scanner manufacturers are summarized in Table 2. Imaging data were obtained using 36 different scanner models from five different manufacturers (GE, *n* = 102; Philips, *n* = 71; Siemens, *n* = 94; Canon/Toshiba, *n* = 60; Fujifilm, *n* = 1).

### 3.2. Diagnostic Performance of AI Application

The overall per-case sensitivity, specificity, accuracy, and AUC of the AI application in the diagnosis of acute CSF were 90.3% (140/155, 95% CI: 84.5–94.5%), 91.9% (159/173, 95% CI: 86.8–95.5%), 91.2% (299/328, 95% CI: 87.5–94.0%), and 0.91 (95% CI: 0.87–0.94), respectively (Table 3). The overall per-case PPV and NPV were 90.9% (140/154, 95% CI: 85.2–94.9%) and 91.4% (159/174, 95% CI: 86.7–94.5%). The MCC was 0.82 which represents a strong predictive performance by the algorithm.

### 3.3. Subgroup Analysis According to Data Sources, Patient Age, and CT Scanner Manufacturers

Table 3 lists the diagnostic performance according to data sources. There were no differences in sensitivity or accuracy among the three data sources, nor in specificity or AUC between the U.S. teleradiology company and the French teleradiology company (all *p* > 0.05).

There were no differences in sensitivity, specificity, accuracy, or AUC across different age groups regardless of the degree of degenerative change (18–45 years, 46–74 years, ≥75 years; all *p* > 0.05) (Table 4). In addition, there were no differences in sensitivity and specificity across CT manufacturers (all *p* > 0.05) (Table 5).

### 3.4. Per-Bounding Box Analysis and Cervical Spinal Level Labeling Validation

**Figure 3:**
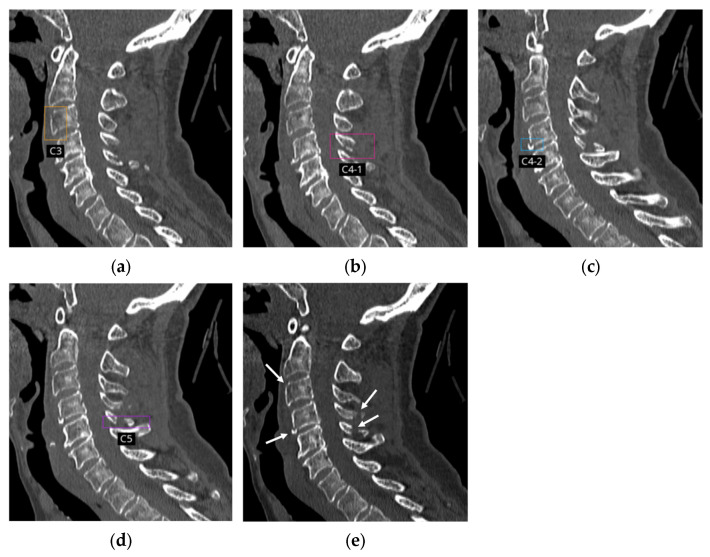
Non-enhanced CT images in a 73-year-old female with four acute fractures of the C3 (vertebral body), C4 (vertebral body and spinous process), and C5 (spinous process), as agreed upon by the first two radiologists. (**a**–**d**) The AI application correctly localized all fractures using bounding boxes and accurately labeled the corresponding spinal levels. Because two fractures were present within C4, the lesions are labeled as C4-1 and C4-2. (**e**) Corresponding sagittal CT image without bounding boxes shows the four fractures (arrows).

The AI application detected 154 positive cases (140 true positive cases and 14 false positive cases). Among these, 23 cases had two bounding boxes, three cases had three bounding boxes, and three cases had four bounding boxes (Figure 3). In total, 192 bounding boxes and 186 vertebral labels were available for analysis. Of the 192 bounding boxes, six (3.1%) did not have an associated vertebral level label because they were located outside the cervical region. Specifically, they were located at the occipital condyle (C0), first thoracic vertebrae (T1) or the junction between two vertebrae (i.e., C1 and C2, or C5 and C6), where vertebral level labeling was not provided by the AI application.

Among the 192 bounding boxes for CSFx localization, there were 30 false positives, resulting in a PPV of 84.4% (162/192, 95% CI: 78.5–89.2%). Of the 186 spina level labels, 181 were correctly labeled, yielding an overall agreement of 97.3% (181/186) for cervical spinal level labeling.

### 3.5. Analysis of Discrepant Cases Between the First Two Radiologists

**Figure 4:**
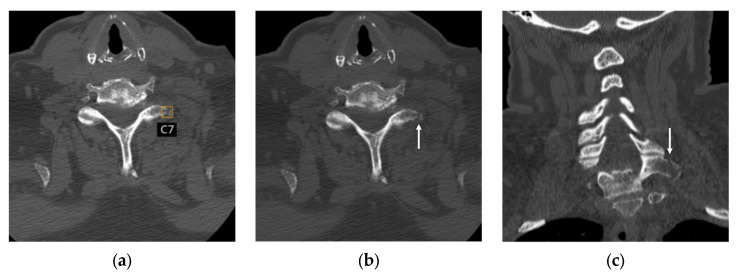
Non-enhanced CT images in a 69-year-old male with an acute fracture of the left transverse process of C7. The first radiologist classified the case as “negative”, whereas the second and third radiologists identified the fracture, representing a discrepant case between the first two radiologists. (**a**) The AI application correctly localized the fracture using a bounding box and accurately labeled the spinal level. (**b**,**c**) Corresponding axial (**b**) and coronal (**c**) CT images without bounding boxes show the fracture of the left C7 transverse process (arrows).

There were 25 cases where the first two radiologists disagreed, meaning one of them misinterpreted the case compared to the reference standard (Figure 4). For these challenging cases, compared to the reference standard, radiologist 1 (excluding one indeterminate case) identified seven true positives, five true negatives, six false positives, and six false negatives. Radiologist 2 (excluding two indeterminate cases) identified seven true positives, six true negatives, five false positives, and five false negatives. The AI application identified seven true positives, eight true negatives, three false positives, and seven false negatives. Thus, radiologist 1, radiologist 2, and the AI application correctly interpreted 12, 13, and 15 cases, respectively.

### 3.6. Analysis of False Positive and False Negative Cases

**Figure 5:**
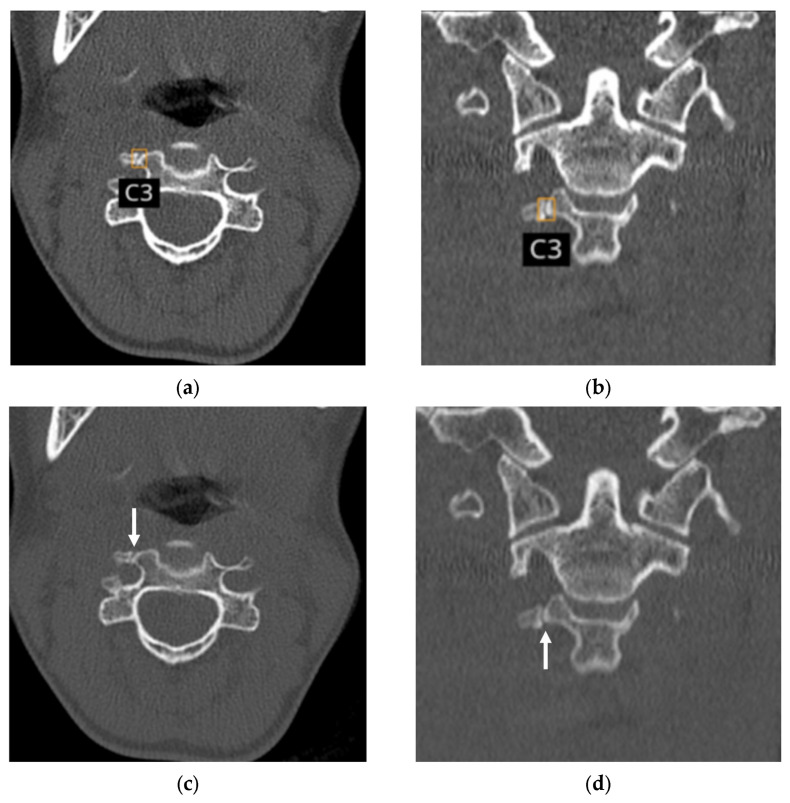
Non-enhanced CT images in a 27-year-old female with a non-acute fracture of the right transverse process of C3. The first radiologist classified the case as “negative”, whereas the second radiologist labeled the non-acute fracture as “positive”, representing a discrepant case between the first two radiologists. The third radiologist classified the case as “negative”. (**a**,**b**) The AI application falsely localized the non-acute fracture, misinterpreting it as acute. The spinal level was correctly labeled. (**c**,**d**) Corresponding axial (**c**) and coronal (**d**) CT images without bounding boxes demonstrate the non-acute fracture of the right C3 transverse process, which is smooth and corticated (arrows).

**Figure 6:**
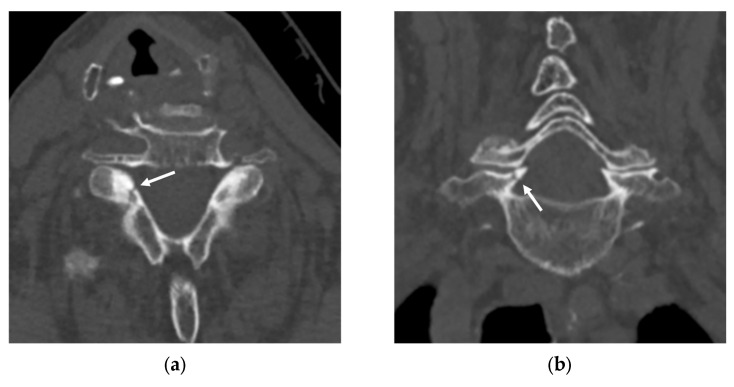
Non-enhanced CT images in an 80-year-old female with an acute nondisplaced fracture of the right superior articular process of C7. The first radiologist classified the case as “negative”, whereas the second and third radiologists identified the fracture, representing a discrepant case between the first two radiologists. The AI application classified the case as “negative”. Axial (**a**) and coronal (**b**) CT images demonstrate an acute nondisplaced fracture of the right C7 superior articular process (arrows).

The AI application demonstrated 14 (4.3%) false positive cases and 15 (4.6%) false negative cases in case-level classification. Three false positive and seven false negative cases were those in which the first two radiologists disagreed (Figure 5 and Figure 6).

Common causes of false positive cases were degenerative changes including osteopenia and osteophytes (*n* = 6), non-acute fractures (*n* = 3), and artifacts including partial volume and motion artifacts (*n* = 3) (Figure 5).

Among the false negative cases, the missed fractures were most commonly located at C2 (*n* = 5), C6 (*n* = 3), and C7 (*n* = 3). Two C2 fractures and one C1 fracture were missed in cases with severe streak and motion artifacts overlapping the fracture site. The majority of missed fractures were subtle, with nine cases presenting as nondisplaced and six cases showing minimal displacement (less than 3 mm) (Figure 6). Regarding fracture dimensions, seven cases measured 1–5 mm in length, and eight cases measured 6–10 mm. Within the 6–10 mm group, seven cases were nondisplaced; the only displaced fracture was a C1 fracture exhibiting 3 mm displacement, which was obscured by prominent streak artifact.

### 3.7. Per-Case Time-to-Notification of AI Application

The mean time to complete per-case classification (positive/negative classification) was 2.9 min (SD: 1.1 min; 95% CI: 2.7–3.0 min). The mean time to complete per-case classification, fracture localization (displaying bounding boxes), and vertebral level labeling was 4.2 min (SD: 1.4 min; 95% CI: 4.1–4.4 min).

## 4. Discussion

This retrospective, multicenter, multinational, and multivendor study evaluated an AI application for detecting acute CSFx, including case-level binary classification, fracture localization, and cervical vertebral level labeling. The overall sensitivity, specificity, accuracy, PPV, NPV, AUC, and MCC of the AI application in the diagnosis of acute CSFx were 90.3%, 91.9%, 91.2%, 90.9%, 91.4%, 0.91, and 0.82, respectively. There were no differences in diagnostic performance in the subgroup analysis by data source, patient age, or CT manufacturer (all *p* > 0.05). Among 192 bounding boxes generated for the 154 cases detected as positive by the AI application, 162 (84.4%) were true positives. Of the 186 bounding boxes with cervical spinal level labels provided by the AI application, 181 were labeled correctly (overall agreement, 97.3%). Among 25 discrepant cases between the first two radiologists, radiologist 1, radiologist 2, the AI application correctly interpreted 12, 13, and 15 cases, respectively.

Compared to previous studies evaluating deep learning models for CSFx detection, the strength of this study is the high performance on a multicenter, multinational, and multivendor dataset, highlighting the potential generalizability of the AI application. Despite the asymmetric prevalence across data sources, the diagnostic performance observed across each source was stable, which strongly supports the generalizability and robustness of the AI model in diverse real-world diagnostic settings Most previous studies of deep learning models for CSFx detection used single-center datasets obtained from single-vendor scanners [5,6,7,9,11], whereas this study validated the AI application on multinational data from diverse hospitals with varying characteristics (a U.S. teleradiology company with higher proportion of trauma cases, a French teleradiology company with fewer trauma and more outpatient cases, and a U.S. university hospital with a level 1 trauma center), using CT scanners from all major vendors. The 2022 RSNA AI challenge data were multinational, multicenter, multivendor data, and the winning algorithm demonstrated higher performance than previous studies [10]. However, the clinical use of the algorithms developed in the RSNA challenge would require regulatory approval and seamless integration in the PACS, which limits their scalability and real-world usability.

The use of a sound reference standard based on the majority vote of multiple radiologists is a key methodological strength of this study, enhancing the reliability of results compared to the previous reports. Establishing a reliable reference standard is a critical first step in accurately evaluating algorithm performance. Previous studies by Ruitenbeek et al. [5] and Voter et al. [7] relied on a comparison of the radiologist report with AI, with only discordant cases undergoing review by another radiologist. Van den Wittenboer et al. [6] and Hu et al. [9] used consensus reading by two radiologists, but for only a subset of the data. While Small et al. [8] and Chen et al. [11] used consensus between two radiologists as the reference standard, the present study is the first to employ a majority vote among multiple radiologists with ROI annotation for localization across the entire dataset.

Our study demonstrated high performance of the AI application in fracture localization and spinal level labeling. To our knowledge, most previous studies focused on case-level classification performance, without evaluating the localization or spinal level labeling. Case-level classification algorithms are designed to prioritize cases for review and improve workflow efficiency. However, false positive results can have the opposite effect, as radiologists may spend additional time searching for non-existent fractures, potentially reducing overall workflow efficiency. By contrast, the high performance of the AI application of this study in fracture localization can increase both confidence in and reliability of case-level classification. In addition, through the review of bounding boxes displayed by the AI application which localize fractures and provide spinal level labels, radiologists can efficiently determine if the AI detected true fractures or false positive lesions and can check the spinal level of the fracture for accuracy.

Despite the high performance of the AI application itself in both case-level classification and localization, additional evidence might be needed to determine whether AI can truly enhance radiologist performance. To assess the impact of AI in complex cases, the 25 cases with disagreement between the first two radiologists were analyzed. In these challenging cases which one of the radiologists misinterpreted, the number of correctly interpreted cases was higher for the AI application than for the two radiologists. This suggests radiologists might decrease the interpretation error with assistance of AI as a second reader, although further reader studies comparing radiologist performance with and without AI assistance are needed.

A large portion of false positive cases in this study were related to the degenerative changes in the spine, as has been reported in previous studies [5,7,8,9]. However, the high sensitivity and specificity seen across age groups in this study demonstrate the robust performance of the AI application. Therefore, physicians can have more confidence with the support of the AI application when interpreting challenging CT images of older patients.

Beyond diagnostic accuracy, real-world clinical deployment requires effective workflow integration. This AI application is designed to operate natively within PACS/RIS, performing background analysis of acute CSFx and enabling triage without altering the standard radiologist workflows. Importantly, fracture localization and spinal level labeling shift AI review from a binary alert to a targeted verification task, which may reduce search time and cognitive load rather than add user burden. Known failure modes (e.g., degenerative changes or image artifacts) are mitigated through intended adjunctive use, with final interpretation remaining under radiologist oversight.

This study has several limitations. First, there is a possibility of spectrum bias due to the retrospective nature of the study. Although data was retrieved consecutively from both teleradiology companies, only positive cases were retrieved from U.S. university hospital. Second, since the primary endpoint of this study was the sensitivity and specificity of the AI algorithm at case-level, only positive cases detected by the AI application were evaluated for localization analysis. Further studies evaluating lesion-level diagnostic performance are needed to enhance reliability and applicability. Third, despite the high performance of the AI application itself, a reader study is needed to prove the added value of the AI application as a second reader. Fourth, the exclusion of cases with discordant or indeterminate diagnoses may have potentially limited AI performance. Although the final reference standard was robustly determined by majority voting in this study, future work would benefit from establishing a reference standard through a consensus meeting or by incorporating accompanying magnetic resonance imaging findings for discordant cases.

In conclusion, the AI application for detecting acute CSFx evaluated in this study demonstrated high diagnostic performance on multicenter, multinational, and multivendor data, with high performance in fracture localization and spinal level labeling. This highlights the AI application’s potential generalizability across variable real-world settings and its ability to improve workflow efficiency.

## Figures and Tables

**Table 1:** Baseline Characteristics of the Study Population.

	U.S. Teleradiology Company (*n* = 176)	U.S. University Hospital (*n* = 22)	French Teleradiology Company (*n* = 130)	*p* Value
Age (y) *	63 ± 20 (19–80)	52 ± 23 (22–90)	48 ± 23 (18–101)	0.02
Age Group				
18–45 years	39 (22.2)	10 (45.5)	64 (49.2)	<0.001
46–74 years	56 (31.8)	6 (27.3)	45 (34.6)	0.62
≥75 years	81 (46.0)	6 (27.3)	21 (16.2)	0.09
Sex				
Male	108 (61.4)	15 (68.2)	72 (55.4)	0.35
Female	56 (31.8)	6 (27.3)	57 (43.8)	0.62
NA	12 (6.8)	1 (4.5)	1 (0.8)	<0.001
Presence of Acute Cervical Spine Fractures				
Positive Cases	127 (72.2)	22 (100)	6 (4.6)	0.002
Negative Cases	49 (27.8)	NA	124 (95.4)	<0.001

Note—Except where indicated, data are the numbers of patients, with percentages in parentheses. *p* values were calculated using the one-way ANOVA for age, and the Chi-square test or Fisher exact for categorical characteristics. NA = not available, * Data are means ± SDs, with ranges in parentheses.

**Table 2:** Distribution of Patients by CT Scanner Manufacturer.

	U.S. Teleradiology Company (*n* = 176)	U.S. University Hospital (*n* = 22)	French Teleradiology Company (*n* = 130)	Total (*n* = 328)
GE	73	0	29	102
Philips	16	22	33	71
Siemens	45	0	49	94
Canon (Toshiba)	41	0	19	60
Fujifilm	1	0	0	1

Note—Data are the numbers of patients.

**Table 3:** Diagnostic Performance of the AI Application by Data Source.

	U.S. Teleradiology Company	U.S. University Hospital	French Teleradiology Company	Overall	*p* Value
Sensitivity (%)	90.6 (84.1, 95.0) [115/127]	90.9 (70.8, 98.9) [20/22]	83.3 (35.9, 99.6) [5/6]	90.3 (84.5, 94.5) [140/155]	0.84
Specificity (%)	89.8 (77.8, 96.6) [44/49]	NA	92.7 (86.7, 96.6) [115/124)	91.9 (86.8, 95.5) [159/173]	0.53 *
Accuracy (%)	90.3 (85, 94.3) [159/176]	90.9 (70.8, 98.9) [20/22]	92.3 (86.3, 96.2) [120/130]	91.2 (87.5, 94.0) [299/328]	0.83
AUC	0.90 (0.85, 0.94)	NA	0.88 (0.812, 0.931)	0.91 (0.87, 0.94)	0.56 *

Note—Data in parentheses are 95% confidence intervals. Data in brackets are raw data. NA = not applicable. * *p* values were calculated for the comparison between the U.S. teleradiology company and the French teleradiology company.

**Table 4:** Diagnostic Performance Metrics of the AI Application by Age Group.

	18–45 Years	46–74 Years	≥75 Years	*p* Value
Sensitivity (%)	90.2 (76.9, 97.3) [37/41]	89.1 (76.4, 96.4) [41/46]	91.2 (81.8, 96.7) [62/68]	0.94
Specificity (%)	91.7 (82.7, 96.9) [66/72]	95.1 (86.3, 99.0) [58/61]	87.5 (73.2, 95.8) [35/40]	0.78
Accuracy (%)	91.2 (84.3, 95.7) [103/113]	92.5 (85.8, 96.7) [99/107]	89.81 (82.51, 94.80) [97/108]	0.98
AUC	0.91 (0.841, 0.955)	0.92 (0.853, 0.964)	0.893 (0.82, 0.945)	0.82

Note—Data in parentheses are 95% confidence intervals. Data in brackets are raw data.

**Table 5:** Diagnostic Performance of the AI Application Stratified by CT scanner manufacturers.

	Sensitivity (%)	Specificity (%)
GE	90.4% (79.0, 96.8) [47/52]	92.0% (80.8, 97.8] [46/50]
Philips	91.2% (76.3, 98.1) [31/34]	91.9% (78.1, 98.3) [34/37]
Siemens	87.2% (72.6, 95.7) [34/39]	90.9% (80.0, 97.0) [50/55]
Canon (Toshiba)	93.3% (77.9, 99.2) [28/30]	93.3% (77.9, 99.2] [28/30]
Fuji film	100.0% (2.5, 100.0) [1/1]	NA
*p* value	0.91	0.98

Note—Data in parentheses are 95% confidence intervals. Data in brackets are raw data. NA = not available.

## Data Availability

The data presented in this study are available on request from the authors due to legal and ethical restrictions by the institutional ethics committee, which do not allow open sharing of clinical and imaging data. De-identified ROI annotations and/or detailed reference-standard process can be shared upon request, pending institutional approval.

## Abbreviations

The following abbreviations are used in this manuscript:

AI	Artificial Intelligence
AUC	Area under the receiver-operating characteristic curve
CSFx	Cervical spine fractures
